# Intraperitoneal cisplatin and doxorubicin as maintenance chemotherapy for unresectable ovarian cancer: a case report

**DOI:** 10.1186/s12885-016-3004-8

**Published:** 2017-01-06

**Authors:** Clemens B. Tempfer, Franziska Hartmann, Ziad Hilal, Günther A. Rezniczek

**Affiliations:** 1 Department of Obstetrics and Gynecology, Ruhr University Bochum, Bochum, Germany; 2 Department of Pathology, Ruhr University Bochum, Bochum, Germany

**Keywords:** Ovarian cancer, Intraperitoneal chemotherapy, Maintenance, Peritoneal carcinomatosis, PIPAC, Quality of life, Antineoplastic agents, Adverse effects

## Abstract

**Background:**

Primary advanced, unresectable ovarian cancer (OC) is treated with palliative systemic chemotherapy. Intraperitoneal chemotherapy may be an alternative local maintenance therapy.

**Case presentation:**

A 75 year old woman with laparoscopically and histologically confirmed unresectable OC was treated with 13 cycles of intraperitoneal cisplatin 7.5 mg/m^2^ and doxorubicin 1.5 mg/m^2^ over 2 years using laparoscopic pressurized intraperitoneal aerosol chemotherapy (PIPAC). Objective tumor response (tumor regression on histology, stable disease on repeated video-laparoscopy and peritoneal carcinomatosis index) was noted. No Common Terminology Criteria for Adverse Events (CTCAE) > grade 3 were observed. EORTC QLQ-C30 quality of life measurements were stable throughout the therapy.

**Conclusions:**

Repeated intraperitoneal chemotherapy with cisplatin and doxorubicin applied as PIPAC may be an effective maintenance treatment in women with primary advanced, unresectable OC.

**Electronic supplementary material:**

The online version of this article (doi:10.1186/s12885-016-3004-8) contains supplementary material, which is available to authorized users.

## Background

Ovarian cancer (OC) usually presents at an advanced stage due to the lack of efficient screening programs and the absence of pathognomonic clinical symptoms. For example, in a large series of 1009 women with epithelial OC, 78% of women initially diagnosed with this disease had Fédération Internationale des Gynécologues et Obstétriciens (FIGO) stage III or IV disease [1]. In women with advanced OC, the tumor typically metastasizes into the abdominal cavity involving the abdominal side walls, the small and large bowel, the mesentery, and the diaphragm. Between 40% and 80% of these women successfully undergo cytoreduction with no residual disease or residual disease <1 cm [2]. However, a substantial proportion of patients is deemed unresectable upon diagnostic laparoscopy or exploratory laparotomy. In these patients, palliative chemotherapy or - alternatively - neoadjuvant chemotherapy with three or four cycles of carboplatin and paclitaxel followed by a second attempt at surgery, i.e. intervention debulking, is the therapy of choice [2, 3]. A number of cytotoxic agents have shown activity in this situation, among them liposomal doxorubicin, topotecan, gemcitabine, and trabectedin [4]. These substances, alone or in combination, achieve a wide range of response rates. For example, Ferrero et al. described a response rate of 49% with gemcitabine and vinorelbine in platinum-sensitive patients [5], whereas Burger et al. found a response rate of 29% in a mixed resistant-sensitive population [6]. Lower response rates are seen among platinum-resistant patients with response rates of 25% [7], 21% [8], 11% [9], and even 3% [10] with doxorubicin and gemcitabine, topotecan and oxaliplatin, gemcitabine and vinorelbine, and vinorelbine in various combinations and dosages, respectively. Regarding survival, palliative chemotherapy regimens achieve median overall survival rates after the first, second, third, fourth, and fifth relapse of 17.6 (95% CI 16.4–18.6), 11.3 (10.4–12.9), 8.9 (7.8–9.9), 6.2 (5.1–7.7) and 5.0 (3.8–10.4) months, respectively [11].

Women with unresectable OC are in a palliative situation and therefore, side effects of toxic chemotherapy and quality of life become important issues when judging the pros and cons of standard treatment regimens. Palliative systemic chemotherapy has a considerable morbidity, especially when given as polychemotherapy with or without targeted therapies such as bevacizumab or olaparib. Common Terminology Criteria for Adverse Events (CTCAE) grade 3 to 4 are seen in up to 50% of patients [4–10]. Thus, effective and less morbid alternatives to systemic palliative chemotherapy are an unmet medical need for women with primary unresectable OC.

Pressurized intraperitoneal aerosol chemotherapy (PIPAC) is a new form of intraperitoneal chemotherapy taking advantage of the physical properties of gas and pressure. PIPAC has been shown to increase the distribution and infiltration depth of intraperitoneal chemotherapy, while at the same time reducing the chemotherapy dose by a factor of 10 as compared with systemic intravenous applications [12]. Furthermore, PIPAC can be administered repeatedly and has been shown to induce regression of peritoneal tumor nodules with limited hepatic and renal toxicity [13]. In a prospective phase II trial of women with recurrent OC, PIPAC with cisplatin and doxorubicin achieved a clinical benefit rate of 62% according to Response Evaluation Criteria In Solid Tumors (RECIST) criteria and a histological tumor regression rate of 76% [13]. In addition, PIPAC has been shown to be well tolerated with a stabilization and even an increase of the quality of life of patients with peritoneal carcinomatosis (PC) from ovarian, gastric, and colon cancer [13, 14].

The documented good tolerability and positive effects on quality of life make PIPAC a candidate for a maintenance chemotherapy in women with unresectable OC. As of yet, however, there are no data describing PIPAC as a possible means of maintenance therapy in patients with primary unresectable OC. Here we report the case of a successful long-term maintenance treatment over two years with intraperitoneal cisplatin and doxorubicin applied as PIPAC.

## Case presentation

We present the case of a 75 year old woman with OC, first diagnosed in 2014. The patient underwent diagnostic laparoscopy with histologic confirmation of a high-grade serous adenocarcinoma of the right ovary. The tumor was not resectable due to extensive small bowel involvement. In addition, due to her clinical condition, the patient declined systemic chemotherapy with carboplatin and paclitaxel. The patient was offered local, intraabdominal chemotherapy with cisplatin and doxorubicin applied as PIPAC as an off-label procedure. The patient provided written informed consent for this treatment and for publication of this case report. We did not obtain Ethics Committee approval, since no approval is required for a singular case report.

From October 2014 to August 2016, the patient underwent 13 cycles q 5–12 weeks of PIPAC with cisplatin 7.5 mg/m^2^ and doxorubicin 1.5 mg/m^2^ at 12 mmHg. PIPAC was performed as described before (5). Briefly, both drugs were applied into the abdomen via laparoscopy using a 12 mmHg CO_2_ pneumoperitoneum, an aerosolizer (Capnopen®, Capnomed, Villingen, Germany), and an intravenous high-pressure injector (Arterion Mark 7, MedRad Bayer Healthcare, Berlin, Germany). A video of a PIPAC application is available in the (Additional file 1: Video S1) of the electronic version of the manuscript. Adverse events were graded according to the CTCAE version 4.0. Quality of life was measured by the standardized European Organization for the Research and Treatment of Cancer (EORTC) QLQ-C30, version 3.0, questionnaire, a validated tool for assessing quality of life in cancer patients. Between October 2015 and May 2016, tamoxifen 20 mg once daily was taken by the patient. In May 2016 intravenous carboplatin area under the curve (AUC) 3 was added on day 2 after each PIPAC due to local progression diagnosed on a computed tomography scan.



**Additional file 1:** with a pressurized intraperitoneal aerosol chemotherapy (PIPAC). A Supplementary Video (41 min) is available on YouTube at: https://www.youtube.com/watch?v=YsRw7Z5ocLY. This video shows the first application of a pressurized intraperitoneal aerosol chemotherapy (PIPAC) performed in the patient described in this case report. Ascites, peritoneal carcinomatosis, adhesions, and application of aerosolized chemotherapy (doxorubicin and cisplatin) via a pressure pump is demonstrated. (WEBM 148 MB)


**Fig. 1:**
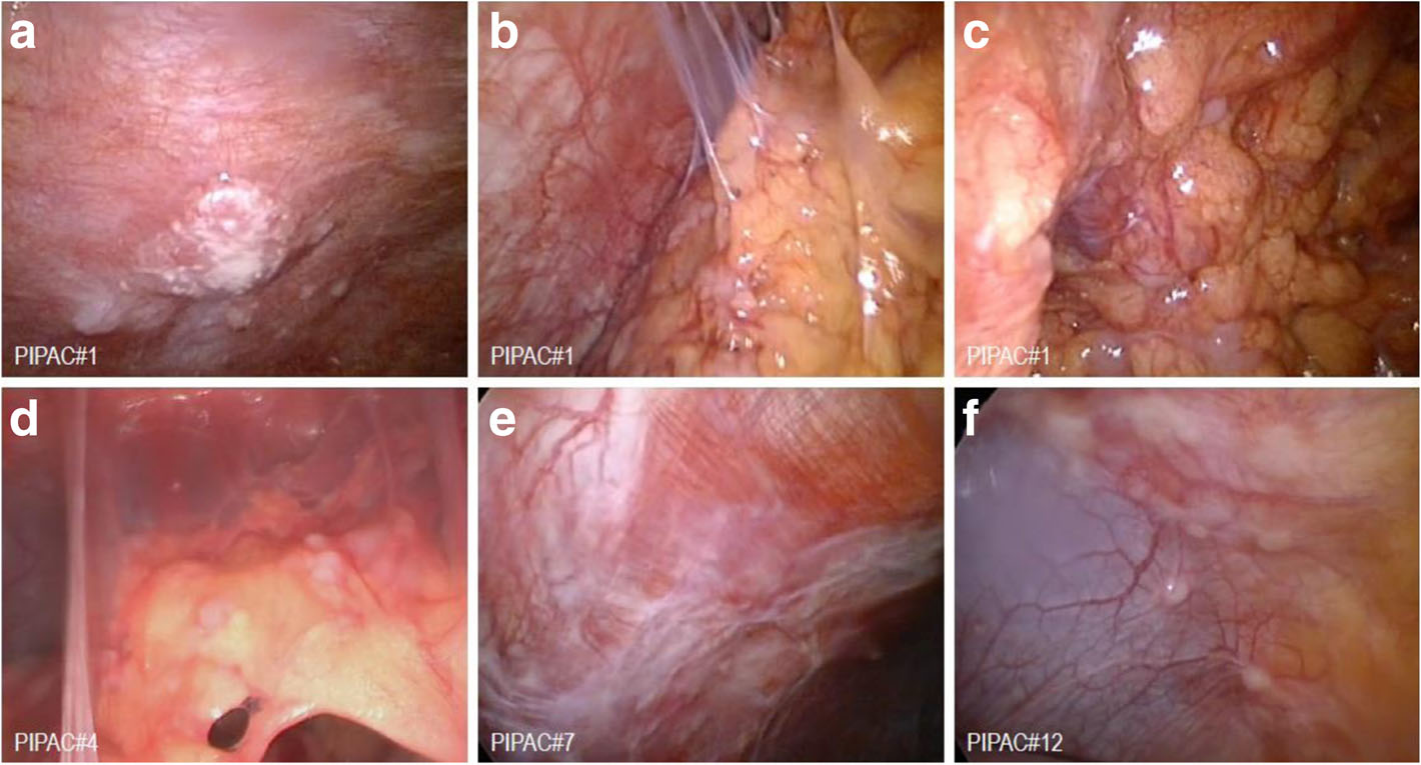
Intraoperative findings (macroscopy) during video-laparoscopy before the first (panels **a**–**c**), fourth (panel **d**), seventh (panel **e**), and twelfth (panel **f**) pressurized intraperitoneal aerosol chemotherapy (PIPAC) application. During the course of therapy, sclerosis and flattening of peritoneal nodules as well as reticular scarring of the visceral and parietal peritoneum were observed

**Fig. 2:**
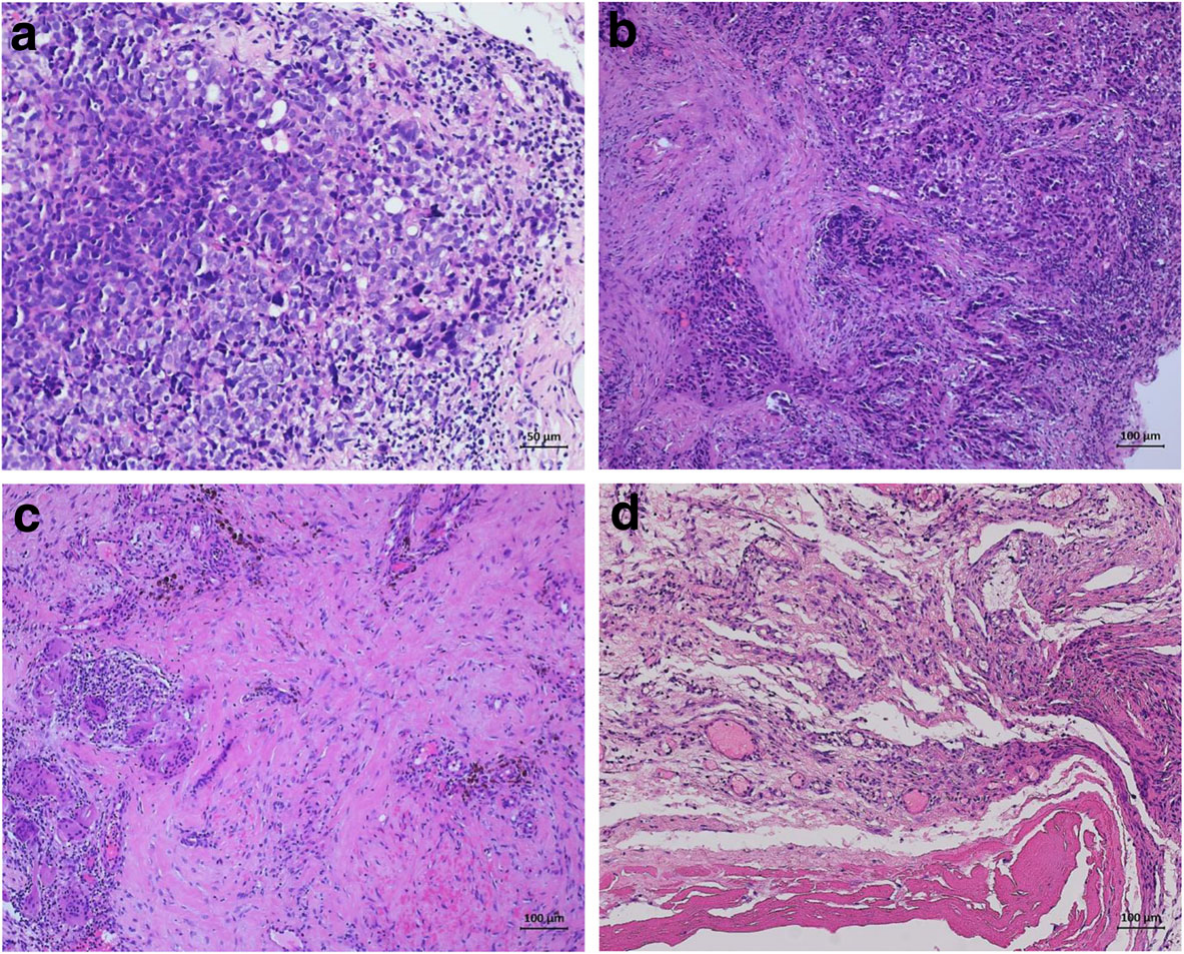
Intraoperative findings (microscopy) before the first pressurized intraperitoneal aerosol chemotherapy (PIPAC) (**a**), and during consecutive PIPAC cycles (PIPAC #3 (**b**), PIPAC #5 (**c**), and PIPAC #6 (**d**). Panel A shows peritoneal manifestations of a serous carcinoma with large, pleomorphic nuclei and frequent mitosis. Histopathological specimens taken during consecutive PIPACs 3, 5, and 6 (panels **b**, **c**, and **d**, respectively) show residual peritoneal tumor foci with reduced cellularity and fibroelastic connective tissue and associated inflammatory changes such as fibrinous exudate, fibrosis, foreign-body reaction, and hemosiderin-laden macrophages. Bars, 100 μm

Figure 1 shows screenshots of video-laparoscopies during consecutive PIPACs demonstrating PC and response to PIPAC, evidenced by fibrosis and reticular sclerosis of PC nodules. Histological objective tumor response was noted by grading of tumor cell regression. Figure 2 demonstrates histopathological specimens of the initial tumor specimen sampled during PIPAC #1 with peritoneal manifestations of serous carcinoma with large, pleomorphic nuclei and frequent mitosis and histopathological specimens taken during consecutive PIPACs with residual peritoneal tumor foci with reduced cellularity and fibroelastic connective tissue with inflammatory changes such as fibrinous exudate, fibrosis, foreign-body reaction, and hemosiderin-laden macrophages.

**Fig. 3:**
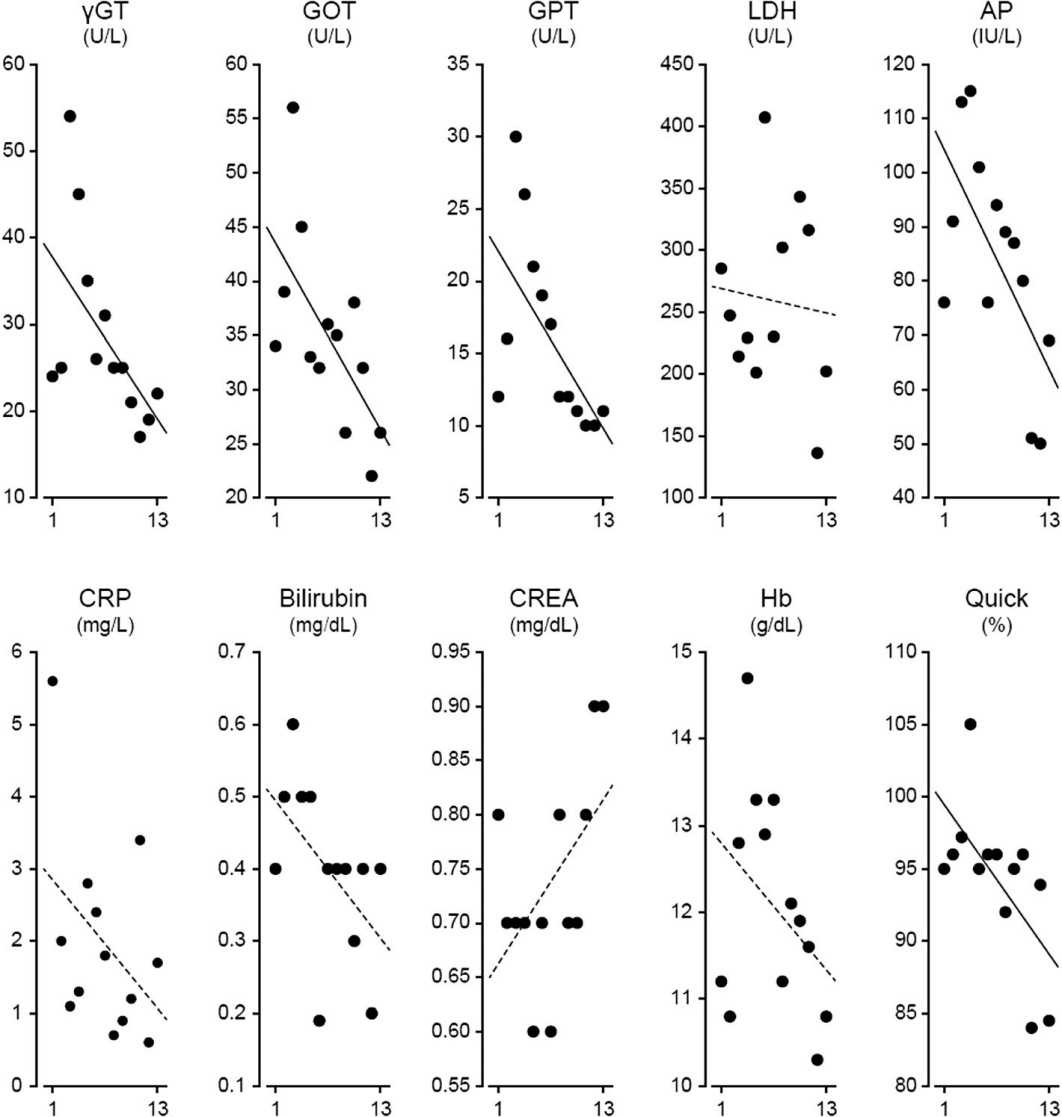
Scatter plots demonstrating serum levels of gamma glutamyl transferase (γGT), aspartate aminotransferase (GOT/ASAT), alanine aminotransferase (GPT/ALAT), lactate (LDH), alkaline phosphatase (ALP), C-reactive protein (CRP), bilirubin, creatinine (CREA), hemoglobin (Hb) and Quick values throughout 13 pressurized intraperitoneal aerosol chemotherapy (PIPAC) procedures. Linear regression lines are shown (solid. Pearson product moment correlation with *p* < 0.05; dotted, no correlation)

**Fig. 4:**
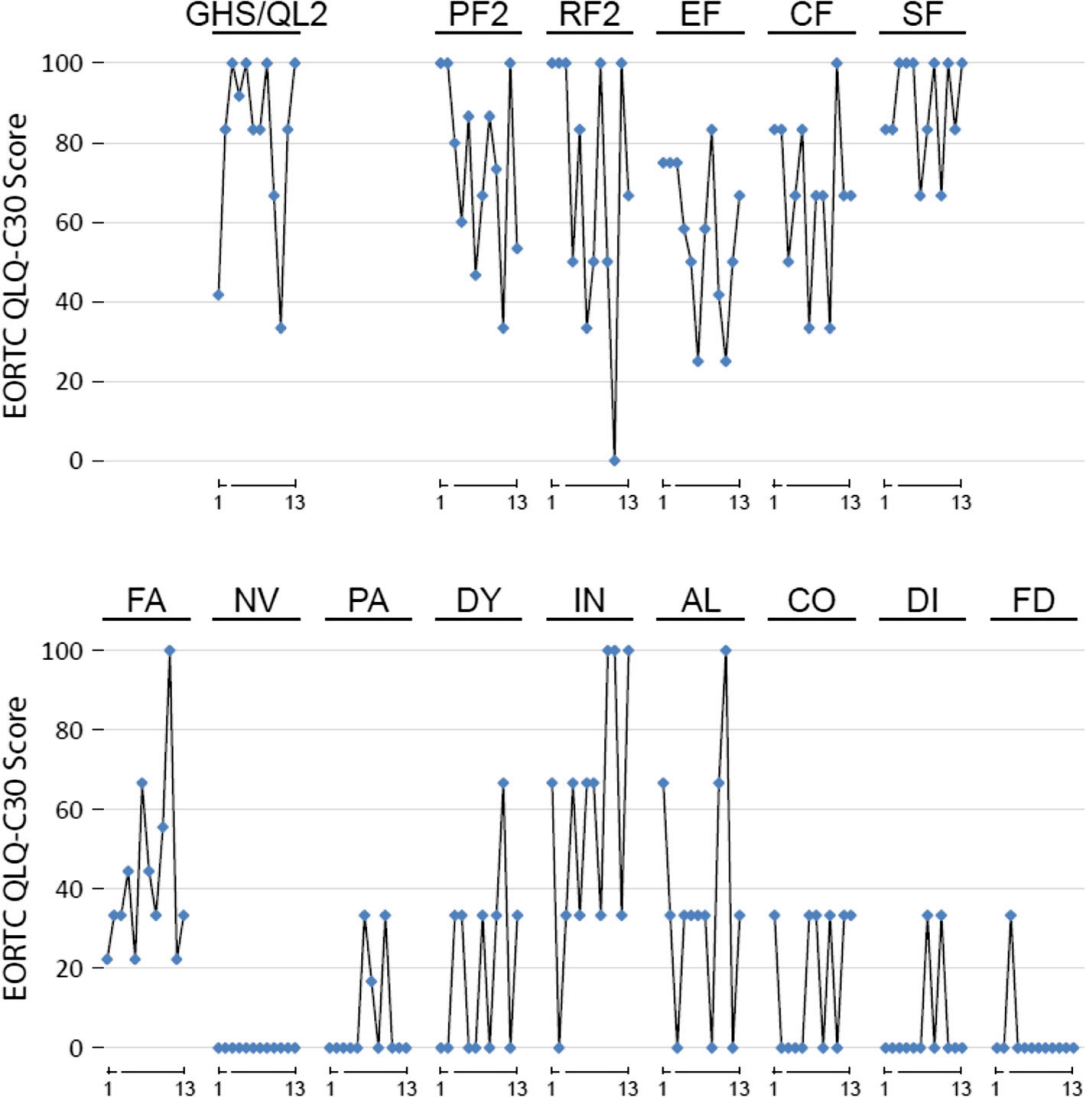
European Organization for Research and Treatment of Cancer (EORTC) QLQ-C30 (version 3.0) scores. GHS/QL2, global health status/QoL (revised). Functional scales: PF2, physical functioning (revised); RF2, role functioning (revised); EF, emotional functioning; CF, cognitive functioning; SF, social functioning. Symptom scales/items: FA, fatigue; NV, nausea and vomiting; PA, pain; IN, insomnia; AL, appetite loss; CO, constipation; DI, diarrhea; FD, financial difficulties. Diamonds represent consecutive pressurized intraperitoneal aerosol chemotherapies (PIPACs, cycles 1–13; note: data for cycle 3 are missing)

Intraoperative assessment of PCI during repeated PIPACs confirmed stable disease. The PCI noted by laparoscopy during the 13 PIPAC sessions was 25, 19, 10, 15, 15, 13, 14, 10, 8, 10, 15, 6, and 12. The treatment was well tolerated. CTCAE events grade 1 (abdominal pain, fever) were noted within 72 h after 7 PIPAC procedures. A CTCAE event grade 3 was observed twice at the time of PIPAC #11 and #12 (two episodes of symptomatic pleural effusions necessitating pleural puncture and drainage). There was no acute or cumulative renal, hepatic, or hematologic toxicity throughout the whole treatment period. We observed stable or declining values of creatinine, γGT, GOT/ASAT, GPT/ALAT, lactate dehydrogenase, alkaline phosphatase, C-reactive protein, bilirubin, and Quick throughout all PIPAC procedures (Fig. 3). Serum levels of CA 125 fluctuated between 1273 and 3486 U/mL. Figure 4 shows EORTC QLQ-C30 quality of life scores indicating a stabilization of the patient’s quality of life during the treatment period. Specifically, scores for pain, vomiting, and obstipation/diarrhea were stable throughout the treatment, suggesting that intraabdominal maintenance chemotherapy with PIPACs does not result in acute or cumulative gastrointestinal toxicity.

## Discussion

Women with primary advanced, unresectable OC are confronted with a palliative situation, which is often characterized by substantial co-morbidities and a low quality of life [4, 15]. The standard of care for these women is systemic polychemotherapy with or without the addition of targeted therapies. The toxicity of these regimens, however, is substantial, making it difficult to balance the advantages of the therapy and the toxicity and loss of quality of life during the limited life expectancy left for these patients. Thus, additional and less toxic treatment options with minimal or no adverse effects on the quality of life are warranted and an unmet medical need at present. Intraperitoneal chemotherapy has been demonstrated to be effective in women with OC in the adjuvant setting. PIPAC is a form of intraperitoneal chemotherapy with minimal systemic side effects, using pressurized, aerosolized chemotherapy in order to improve drug distribution and tumor penetration [12]. Preliminary data in patients with advanced PC demonstrate good tolerability and objective tumor response [12, 13]. PIPAC can be applied repeatedly without cumulative toxicity and a low amount of systemic chemotherapy burden. Previous studies have demonstrated that PIPAC does not adversely affect quality of life [13, 14]. These properties make PIPAC a potential alternative to systemic chemotherapy in women with primary advanced, unresectable OC.

We present the case of a 75 year old woman with unresectable OC and the successful application of 13 cycles of PIPAC as maintenance therapy over a period of two years. Objective tumor response was noted, defined as tumor regression on histology and video-laparoscopy. Clinical assessments, serum CA 125 measurements, and consecutive PCI values showed stable disease. Most importantly, quality of life did not decline and was maintained at a high level throughout the therapy. This case report suggests that PIPAC may be a reasonable alternative to systemic chemotherapy in selected women with primary advanced, unresectable OC.

Besides ovarian cancer, PIPAC has been tested in other peritoneal surface malignancies, e.g. gastric cancer [14], colorectal cancer [16], pseudomyxoma peritonei [17], suggesting that this is an effective tool for the treatment of various forms of PC using different drugs such as oxaliplatin, cisplatin, and doxorubicin. The goold local and systemic tolerability of PIPAC as suggested by this case report and a series of other studies [12–14, 16, 17], indicates that more drugs and alternative combinations of drugs should be tested via the PIPAC approach.

QoL is a major treatment goal in oncology, especially in palliative patients. Thus, we made efforts to document eventual changes in QoL throughout the treatment using a validated tool, i.e. the EORTC QLQ-C30 questionnaire. The treatment was well tolerated and the QoL initially improved during therapy with an intermittent decline after 10 treatment courses. Cumulative hepatic or renal toxicity was not observed. Also, gastrointestinal QoL measures remained stable throughout all PIPAC courses, which indicates that PIPAC, although repeatedly applied directly into the abdomen, does not result in acute or cumulative gastrointestinal toxicity.

It has to be stated, however, that this patient could have also achieved stable disease with conventional chemotherapy without the costs and burden of 13 serial laparoscopies. Therefore, PIPAC may be an alternative to systemic chemotherapy fpor patients refusing systemic chemotherapy, but willing to undergo intraperitoneal chemotherapy.

## Conclusion

PIPAC is a new form of IPC, which can be applied repeatedly over a long period of time and with a minimal impact on quality of life. PIPAC may be a reasonable alternative to systemic chemotherapy in selected women with primary advanced, unresectable OC given that it preserves quality of life and has minimal systemic side effects.

## Abbreviations

CTCAE: Common Terminology Criteria for Adverse Events
EORTC: European Organization for Research and Treatment of Cancer
FIGO: Fédération Internationale des Gynécologues et Obstétriciens
GOT/ASAT: glutamate oxalacetate transferase/aspartate aminotransferase
GPT/ALAT: glutamate pyruvate transaminase/alanine aminotransferase
OC: Ovarian cancer
PIPAC: Pressurized intraperitoneal aerosol chemotherapy
QLQ: Quality of life
RECIST: Response Evaluation Criteria In Solid Tumors
γGT: γ-glutamyl transferase
